# Post-marketing drug safety surveillance of enfortumab vedotin: an observational pharmacovigilance study based on a real-world database

**DOI:** 10.3389/fimmu.2024.1397692

**Published:** 2024-08-20

**Authors:** Mingming Yu, Lijun Zhou, Mengda Cao, Chunmei Ji, Yuanyi Zheng

**Affiliations:** ^1^ Department of Ultrasound in Medicine, Shanghai Jiao Tong University Affiliated Sixth People’s Hospital, Shanghai, China; ^2^ Department of Urology, Shanghai Children’s Hospital, Shanghai Jiao Tong University, Shanghai, China; ^3^ Department of Pharmacy, Zhongda Hospital, School of Medicine, Southeast University, Nanjing, China; ^4^ Department of Pharmacy, First Affiliated Hospital of Nanjing Medical University, Nanjing, Jiangsu, China

**Keywords:** enfortumab vedotin, FDA Adverse Event Reporting System, real-world, disproportionality analysis, adverse event

## Abstract

**Background:**

Enfortumab vedotin (EV) is an antibody-drug conjugate (ADC) that has been approved by the FDA for patients with locally advanced or metastatic urothelial carcinoma (UC). This study presents a comprehensive pharmacovigilance analysis of the post-marketing safety profile of EV in the real-world based on the US Food and Drug Administration Adverse Event Reporting System (FAERS).

**Methods:**

Adverse event (AE) reports regarding EV between January 2020 and December 2023 were obtained from the FAERS database. The standardized MedDRA query (SMQ) narrow search AEs on the preferred term (PT) level were used. Disproportionality analysis was performed to identify the AE signals for EV with the reporting odds ratio (ROR), proportional reporting ratio (PRR), multi-item gamma Poisson shrinker (MGPS), and Bayesian confidence propagation neural network (BCPNN).

**Results:**

A total of 2,216 reports regarding EV were included in the present study. SMQ analysis results indicated that a stronger strength signal was found in severe cutaneous adverse reactions, retroperitoneal fibrosis, and peripheral neuropathy. A total of 116 significant disproportionality PTs referring to 14 system organ classes (SOCs) were retained by disproportionality analysis, with 49 PTs not listed on the EV drug label. Frequently reported EV-related AEs included rash, peripheral neuropathy, decreased appetite, alopecia, and pruritus. The time to onset of the majority of EV-related AEs was within 30 days (66.05%), with only 0.73% events occurring after 1 year.

**Conclusion:**

The disproportionality analysis highlights that dermatologic toxicity and peripheral neuropathy were the major AEs induced by EV. The potential AEs not listed on the drug label were mainly related to gastrointestinal, hepatic, and pulmonary events. Further research is needed to confirm and explore the EV-related AEs in clinical practice.

## 1 Introduction

Urothelial carcinoma (UC) is the most common malignancy in the urinary system, has poor long-term survival, and is the predominant histological subtype of bladder cancer (BC) (1). Despite notable advancements in the management of early-stage disease, the treatment of UC remains challenging. Currently, the standard approach and first-line treatment for patients with inoperable locally advanced or metastatic UC is cisplatin-based combination chemotherapy (2–4). However, a considerable number of patients failed to respond or progress after an initial response, which highlights that there is an unmet need for new therapeutic options.

Antibody-drug conjugates (ADCs), which can deliver potent cytotoxic drugs selectively to antigen-expressing tumor cells by linking cytotoxins to monoclonal antibodies (mAbs), present a promising alternative therapeutic option for UC patients (5). Extensive clinical trials have explored the potential of using multiple ADCs in the treatment of UC, including targeting antigens such as SLITRK6, HER-2, EpCAM, and TF-011. The clinical trials data indicated that these ADC drugs were effective in UC, particularly in advanced stages of the disease. Among these ADC drugs, two ADCs have been utilized in UC with clinical significance, enfortumab vedotin (EV) and sacituzumab govitecan (SG), which were the only two FDA-approved ADCs for UC treatment. EV was the first FDA-approved ADC for the treatment of locally advanced or metastatic UC.

EV is a fully humanized IgG1 antibody that incorporates a microtubule-disrupting agent (monomethyl auristatin E, MMAE) and is coupled by a protease-cleavable linker to an mAb targeting nectin-4 (6). EV binds to nectin-4-expressing cells, followed by internalization of the ADC-nectin-4 complex and the release of MMAE via proteolytic cleavage. The release of MMAE disrupts the microtubule network within the cell, ultimately inducing cell cycle arrest and apoptotic cell death (7–9). The EV was approved for adult patients with locally advanced or metastatic UC who had previously received a programmed death receptor-1 (PD-1) or programmed death-ligand 1 (PD-L1) inhibitor and platinum-containing chemotherapy in the neoadjuvant/adjuvant locally advanced or metastatic setting by the FDA in December 2019. This approval was based on the clinical trial EV-201 (NCT03219333) (10, 11). In subsequent years, additional clinical trials further verified the efficacy of EV in locally advanced or metastatic UC patients who have progressed after chemotherapy or immunotherapy (12–14). All of these clinical trials findings confirmed EV as a treatment option in patients previously treated with locally advanced or metastatic UC. Furthermore, the international guidelines (NCCN, EAU, and CUA) recommend EV as a second-line agent in cisplatin-ineligible patients who progressed after PD-1/PD-L1 blocker treatment and as a third-line agent in patients who progressed after receiving chemotherapy and PD-1/PD-L1 blocker treatment (4, 15).

According to Cancer statistics, 2023, there are approximately 82,290 new cases of urinary bladder cancer in the US every year (16). Research findings, national guidelines, and growing clinical experience may be leading to more prescriptions of EV. Thus, it is crucial to understand the safety profile of EV in clinical practice. Clinical trials reported that the most common AEs for EV were fatigue, peripheral neuropathy, decreased appetite, rash, alopecia, nausea, dysgeusia, diarrhea, dry eye, pruritus, and dry skin. Noteworthy toxicities include hyperglycemia, infusion-site extravasation, ocular disorders, peripheral neuropathy, and skin reactions (10, 12, 14, 17). However, there may be some differences for EV-related AEs in clinical trials and clinical practice, due to the fact that clinical trials are conducted under conditions that are not completely equivalent to the real world. This study investigated the comprehensive safety signals of EV in the real world through disproportionality analysis in the hope of providing valuable safety evidence for EV in clinical practice.

## 2 Materials and methods

### 2.1 Data source and processing

This study performed a disproportionality analysis based on the US Food and Drug Administration Adverse Event Reporting System (FAERS), which is a publicly available database of adverse event (AE) reports submitted to the FDA by patients, healthcare professionals, and pharmaceutical companies. FAERS is a useful tool for the post-marketing safety surveillance of drugs and can be employed to assess the potential association between drugs and AEs.

The FAERS data files can be extracted from the website (https://fis.fda.gov/extensions/FPD-QDE-FAERS/FPD-QDE-FAERS.html). This study used data from the first quarter of 2020 (2020Q1) to the fourth quarter of 2023 (2023Q4) because the FDA approved EV for locally advanced or metastatic UC patients on December 18, 2019. All AE reports associated with EV were filtered out by searching with generic and brand names (enfortumab vedotin-ejfv, enfortumab vedotin, PADCEV™), and only reports documenting EV as “primary suspect (PS)” drugs were retained. Otherwise, the duplicate reports in the FAERS database were removed according to the FDA guidelines. When the CASEID were the same, the latest FDA_DT reports were selected; when FDA_DT and CASEID were the same, the higher PRIMARYID reports were chosen.

The AEs in the FAERS database were standardized and classified with preferred terms (PTs) and systematic organic classification (SOC) by the Medical Dictionary for Regulatory Activities (MedDRA) (English version 26.1). Furthermore, different PTs are grouped with standardized MedDRA queries (SMQs), which provide meaningful broader categories representing specific medical conditions or areas of interest. The hierarchical and multi-axial structure of MedDRA provides flexibility in retrieving AEs. To investigate the distribution of EV-related PTs, SMQs were screened by a “narrow” version.

The time to onset of AE reports were assessed. The time to onset was defined as the interval between the date of occurrence of adverse events (EVENT_DT) and the start date of EV administration (START_DT). In addition, the missing and errors data were excluded, such as an earlier EVENT_DT than START_DT.

### 2.2 Data mining

**Figure 1:**
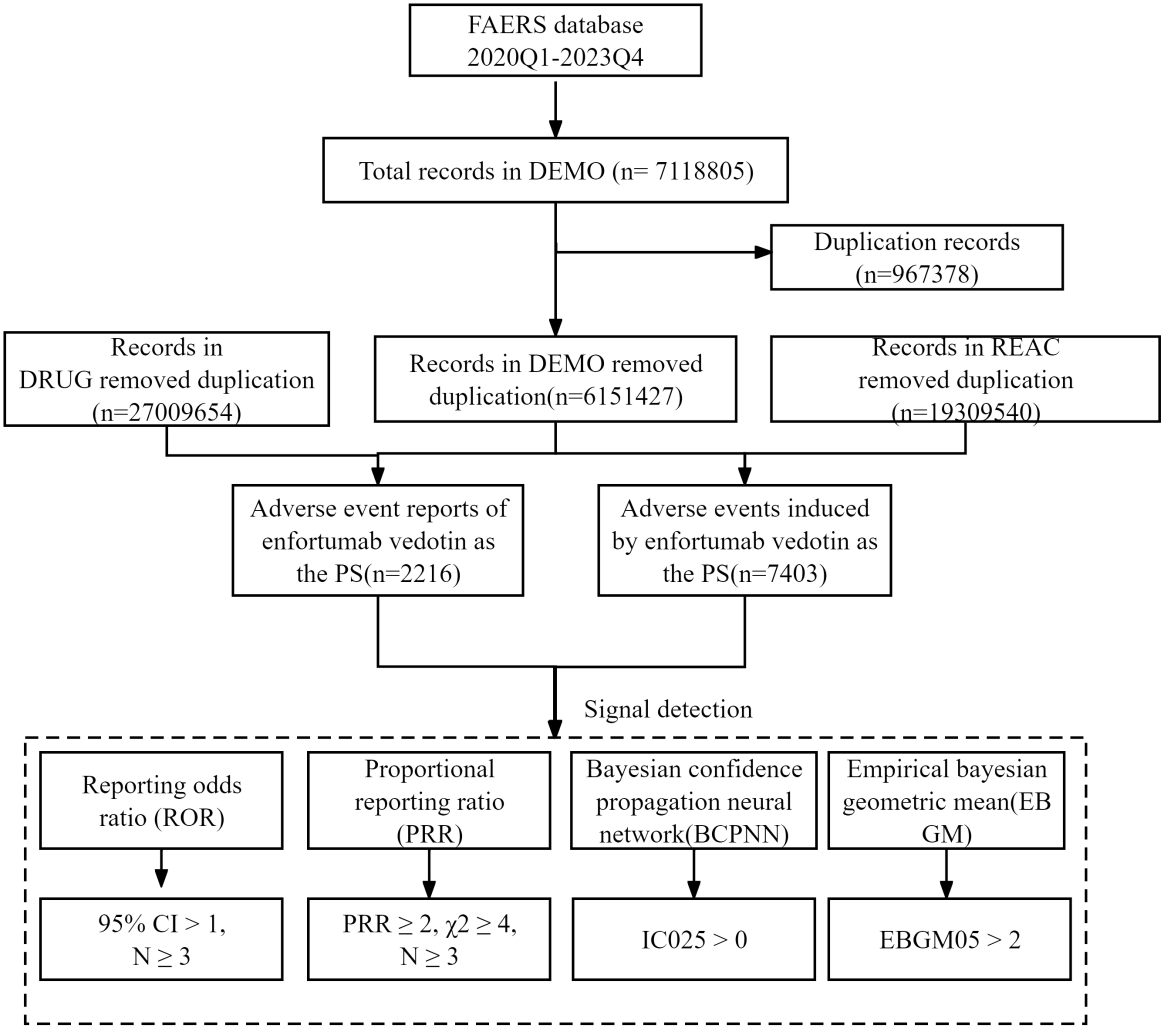
Flow diagram of the selection of enfortumab vedotin-related AEs from the FAERS database.

Disproportionality analysis was performed to detect the potential AE signals of EV using four algorithms: reporting odds ratio (ROR), proportional reporting ratio (PRR), Bayesian confidence propagation neural network (BCPNN), and empirical Bayesian geometric mean (EBGM), which were based on a two-by-two contingency table (**Supplementary Table 1**) (18–20). The specific formulas for the four algorithms and criteria of positive signals are summarized in **Supplementary Table 2**. A positive signal was determined when the AE signals met all four algorithm criteria. Otherwise, some PTs might be reported at high rates with EV when they were actually associated with the underlying disease and not the drug. To avoid interference, EV-related indications (e.g., malignant metastases, malignancy-related morbidities, and malignancy progression) and not drug-related AEs (e.g., product issues and social circumstances) were excluded. Furthermore, the positive signals were meticulously compared with the drug label to identify any signals that were not listed on the drug label. All data processing and statistical analysis were performed using MySQL and Microsoft Excel. The detailed research procedure is depicted in **Figure 1**.

## 3 Results

### 3.1 General characteristics

From 1 January 1 2020 to 31 December 2023, approximately 7,118,805 records were submitted to the FAERS database. After removing duplicate records according to the FDA’s recommended method, 6,151,427 records were selected. Finally, a total of 2,216 records that took the EV as the suspected drug were included. The detailed data processing procedure is shown in **Figure 1**.

The clinical characteristics of AE reports that took EV as the suspected drug in the FAERS database are listed in **Table 1**. The included patients were predominantly male (74.68%), and patients aged >45 constituted the vast majority of AE reports (70.93%). The reports were mainly submitted by physicians (42.78%) and consumers (34.93%), which were mainly from Japan (44.00%) and America (37.32%). The majority of the reports noted one or more outcomes due to the industry reporting requirements. Most EV-related AE reports were associated with a hospitalization-initial or prolonged outcome (770, 34.70%), and approximately 446 patients suffered death (20.10%), which may due to the progression of cancer. 76.99% records were reported in 2022 and 2023, which indicated an increasing clinical use of EV in recent years.

**Table 1:** Characteristics of adverse event reports taking enfortumab vedotin as a suspected drug in the FAERS database (1 January 2020 to 31 December 2023).

Characteristic	Number (n)	Proportion (%)
Number of reports	2,216	
Sex		
Male	1,655	74.68
Female	480	21.66
Unknown	81	3.66
Age (year)		
<45	21	0.95
45-64	291	13.13
65-74	590	26.62
≥75	691	31.18
Unknown	623	28.11
Outcomes		
Hospitalization-initial or prolonged (HO)	770	34.70
Disability (DS)	25	1.10
Life-threatening (LT)	120	5.40
Death (DE)	446	20.10
Reporter’s type		
Physician (MD)	948	42.78
Pharmacist (PH)	179	8.08
Health Professional(HP)	301	13.58
Consumer (CN)	774	34.93
Unknown	14	0.63
Reporter country (top three)		
Japan (JP)	975	44.00
America (US)	827	37.32
France (FR)	99	4.47
FDA received initial case data		
2020	208	9.39
2021	302	13.63
2022	854	38.54
2023	852	38.45

### 3.2 Disproportionality analysis

**Figure 2:**
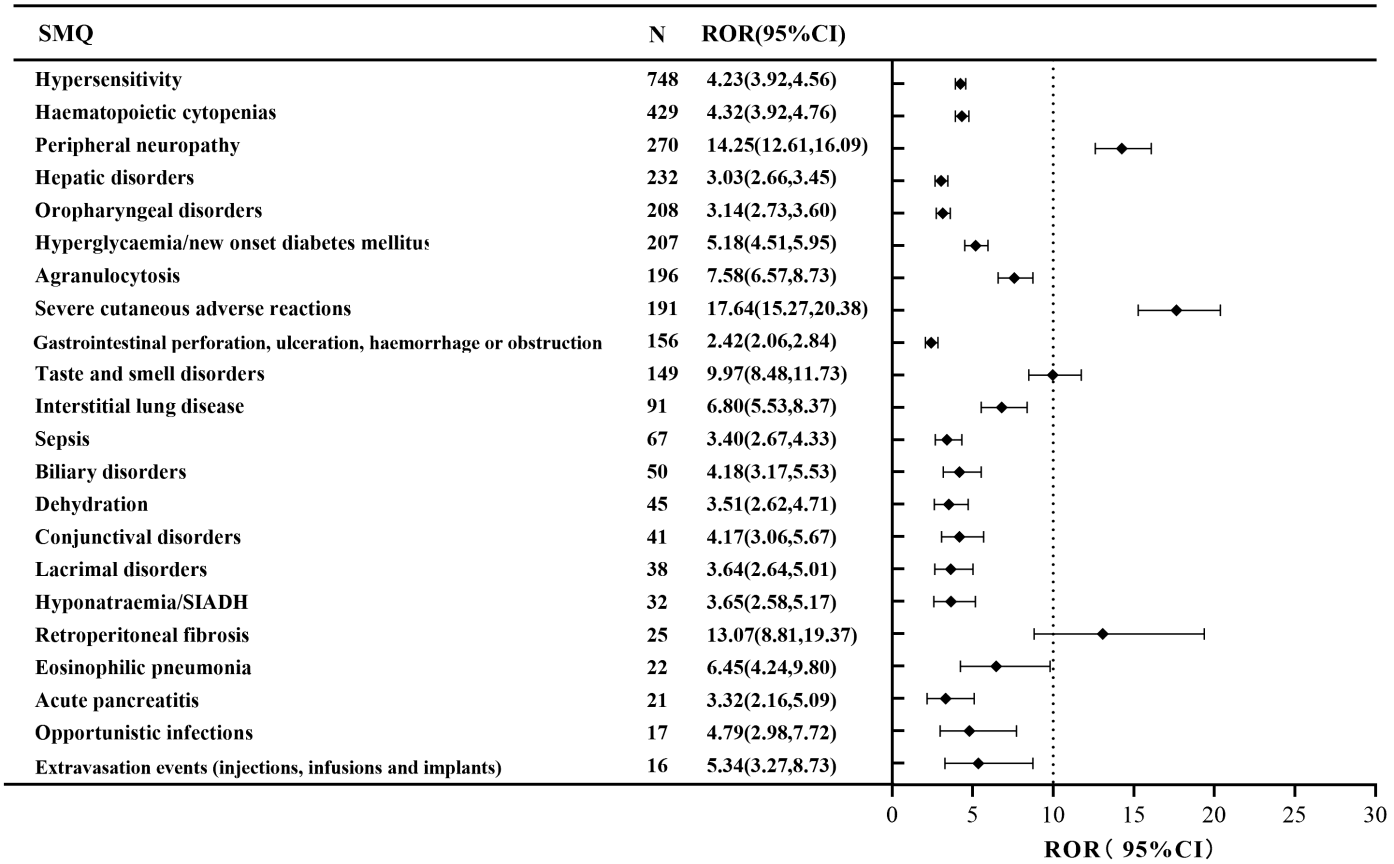
The positive signal distribution of enfortumab vedotin using standardized MedDRA queries at the SMQ level.

A narrow SMQ search was conducted and proceeded with signal detection to comprehensively discover specific clinical cases related to AEs reported for EV. The results indicated that EV was involved in 22 statistically significant SMQs (**Figure 2**). The SMQ “hypersensitivity” occupied the highest percentage of reports, followed by “hematopoietic cytopenias” and “peripheral neuropathy”. Furthermore, severe cutaneous adverse reactions (ROR, 17.64), retroperitoneal fibrosis (ROR, 13.07), and peripheral neuropathy (ROR, 14.25) had higher signal strength. Detailed information on the signal strength for EV based on the SMQ level is shown in **Supplementary Table 3**.

**Figure 3:**
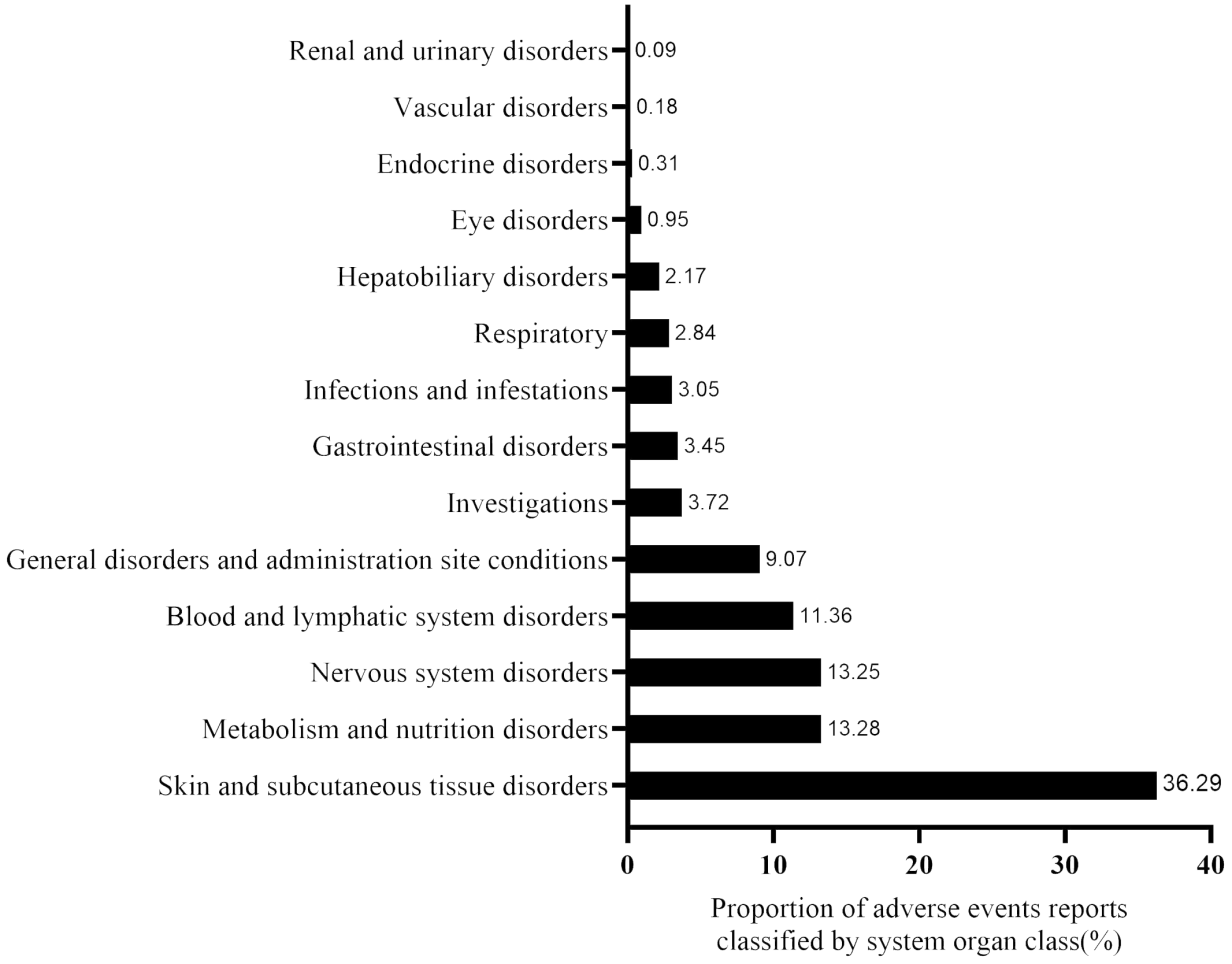
The proportion of enfortumab vedotin-related AE reports classified by system organ class (SOC).

**Figure 4:**
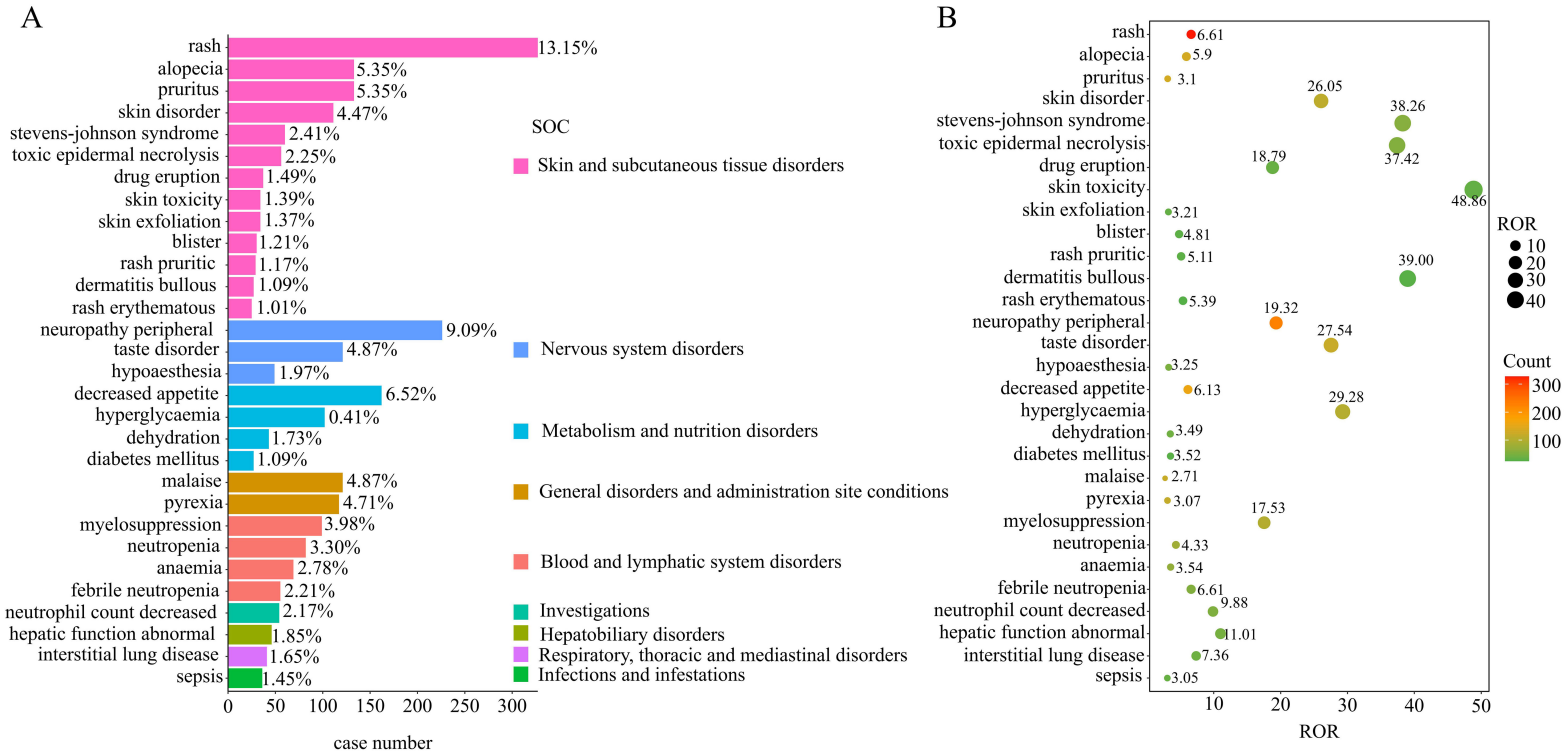
Disproportionality analysis of enfortumab vedotin. **(A)** Bar plot shows the enfortumab vedotin-related AE report numbers of the top 30 PTs. The color indicates the SOC of the corresponding PT. **(B)** Bubble chart shows the ROR of the top 30 PTs.

The disproportionality analysis detected 116 significant disproportionality PTs, which were found to meet all the four calculation criteria simultaneously (**Supplementary Figure 1**). The 116 PTs were referred to 14 SOCs; the AE distribution based on SOC level is displayed in **Figure 3**. The AEs were predominantly of PTs, with the majority belonging to SOCs such as “skin and subcutaneous tissue disorders” (36.29%), “metabolism and nutrition disorders” (13.27%), and “nervous system disorders” (13.24%). The detail distribution of positive signals of EV based on PTs categorized by SOCs are showed in **Supplementary Table 4**. Furthermore, the most frequently reported PTs were rash (n=327; ROR, 6.61), peripheral neuropathy (n=226; ROR, 19.32), decreased appetite (n=162; ROR, 19.32), alopecia (n=133; ROR, 5.90), and pruritus (n=133; ROR, 3.10). The information for the SOC, report number, and ROR of the top 30 PTs are displayed in **Figure 4**. The signal strengths for all EV-positive signals based on PTs categorized by SOCs are described in **Supplementary Table 5**.

Among these significant PTs, 49 were not listed on the drug label, which mainly focused on skin and subcutaneous tissue disorders, gastrointestinal disorders, metabolism and nutrition disorders, and hepatobiliary disorders in the SOC level. Some significant unlabeled PTs with high RORs and report numbers were revealed, including Stevens–Johnson syndrome (SJS) (n=60; ROR, 38.26), toxic epidermal necrolysis (n=56; ROR, 37.42), and hepatic function abnormal (n=46, ROR=11.01); the detail information is described in **Supplementary Table 5**.

### 3.3 Time-to-onset analysis

**Figure 5:**
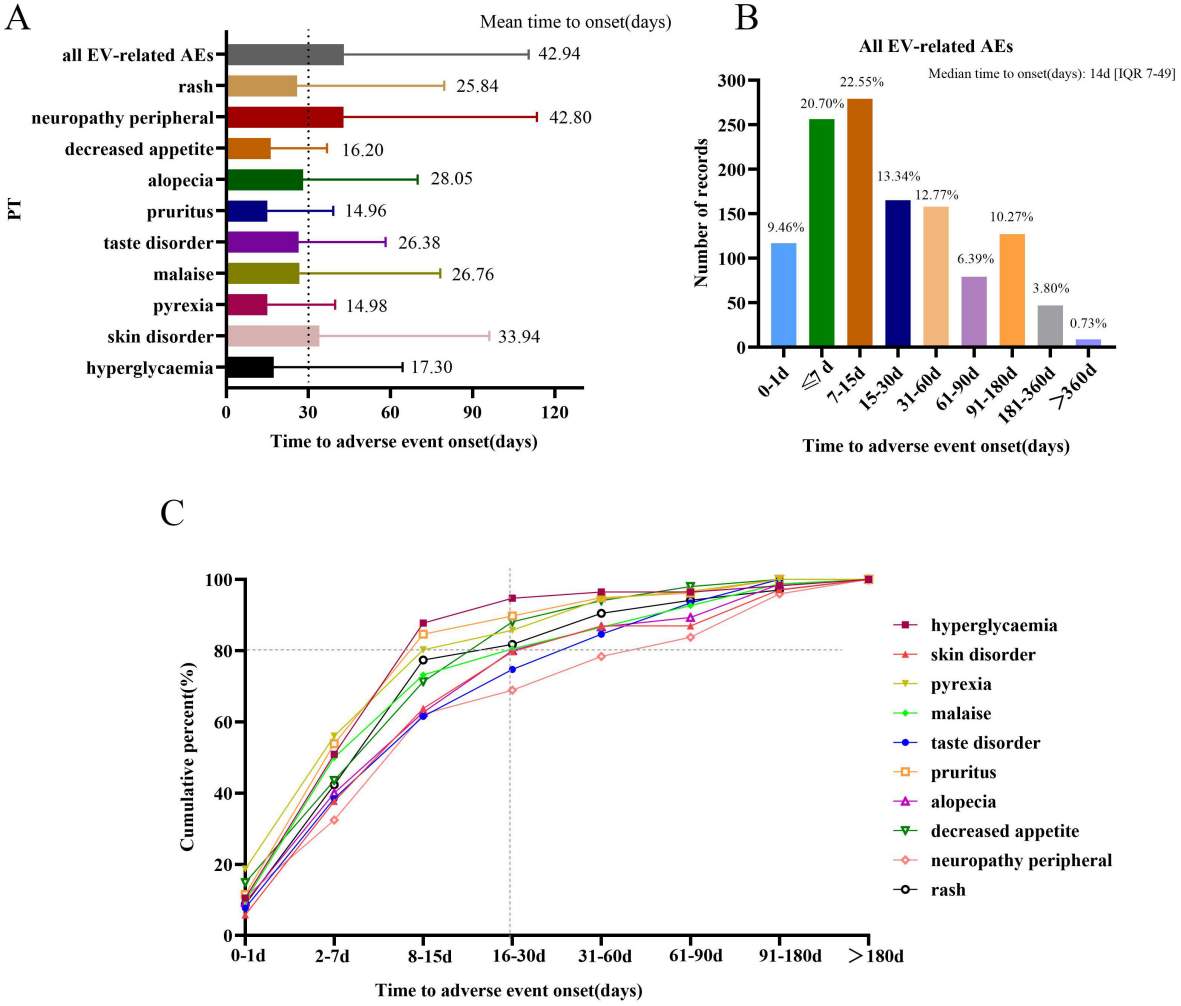
Time to onset of AEs. **(A)** Time of onset of all enfortumab vedotin-related AEs and the top 10 most frequently reported preferred terms. **(B)** The number of reports for all enfortumab vedotin-related AEs in different periods. **(C)** The cumulative percentage of the top 10 most frequently reported preferred terms in different periods.

Approximately 1,237 EV-related AE reports were extracted from the FAERS database, which reported both EVENT_DT and START_DT. The mean onset time of total all EV-related AEs and the 10 most frequently reported AEs are shown in **Figure 5A**. The mean onset time for total all EV-related AEs was 42.94 ± 67.47 days and the median onset time was 14 days [interquartile range (IQR)7, 49 days]. The majority of AEs occurred within 30 days of administration with EV (n=817, 66.05%); less than 1% of AEs occurred after 1 year (**Figure 5B**). Overall, **Figure 5A** shows that the mean onset time of the 10 most frequently reported PTs was less than 30 days, except skin disorder (33.94 days) and peripheral neuropathy (42.80 days). The cumulative percentages of AE reports occurring within 30 days for the top 10 frequently reported PTs were above 80%, except for peripheral neuropathy (68.92%), taste disorder (74.73%), and skin disorder(79.71%) (**Figure 5C**).

## 4 Discussion

The therapeutic landscape for UC management has changed significantly over the last decade. For metastatic UC patients who progressed on platinum-based chemotherapy and immunotherapy, EV has proven to be efficacious and safe for UC patients in clinical trials (EV-101,EV-201,EV-301) (10, 12–14, 21–23). EV has changed the standard of care treatment with clinical significance in UC patients, leading to its widespread use in clinical practice. Thus, the potential AEs for EV in the real world have become a matter of great concern. The present study performed a vigilance study to identify positive AE signals of EV by disproportionality analysis based on the FAERS database.

Our analysis revealed that the majority of positive AE signals belonged to skin disorders, which presented as erythematous scaly pruritic papules and so on. Among these skin disorder AE-related PTs, some were severe cutaneous AEs, including SJS and toxic epidermal necrolysis (TEN). Our SMQ analysis indicated that severe cutaneous adverse reactions were a statistically significant signal. Clinical trials also indicated that skin reactions were frequent and anticipated AEs for EV, and most skin reaction cases were grade 1 or 2; reports described severe events including life-threatening SJS/TEN cases (24–26). SJS/TEN are known AEs of EV in premarketing trials and already included in the boxed warning of the EV manufacturer’s US labeling (27). In this article, we discovered that 93 patients receiving EV experienced SJS/TEN in the FAERS database from the FDA approval of EV between January 2020 and December 2023. Of these patients, 58 died. Unfortunately, the cause of death could not be determined by the FAERS data, which may due to the disease progression of cancer or other factors. Yang et al. also reported that a significant signal was detected between EV and cutaneous toxicities; SJS and TEN were significantly associated with EV use (28). Clinicians should be aware of the EV-related dermatologic toxicities and implement early identification. Thus, it is crucial to maintain close monitoring and vigilance for skin reactions, particularly SJS/TEN in clinical practice. Dermatologic toxicities frequently require therapy interruption, dose reduction, and/or discontinuation, and the appropriate therapy is chosen depending on the severity of the AEs (25, 29). Currently, the mechanism for dermatologic toxicity with EVs is still unknown, and some studies believe that MMAE may be an underlying cause of dermatologic toxicity. Other ADC drugs containing MMAE have also been observed to have dermatologic toxicity (30). Alternatively, nectin-4 is expressed in the skin and plays a role in cell-cell attachment. Thus, disruption of nectin-4 by EV might lead to dermatologic toxicity (25, 31, 32).

Other than dermatologic toxicity, peripheral neuropathy was also reported frequently in the FAERS database (n=226). Peripheral neuropathy (SMQ) also had a higher signal strength. The results were largely consistent with the published study, which reported that the incidence of peripheral neuropathy in ADCs was 39.6% (33). The underlying mechanism might be related to MMAE, which disrupts microtubules (MTs) and causes neurodegeneration (34). Masters et al. (35) included 70 publications and found that peripheral neuropathy was most common in ADCs with an MMAE payload (35). Peripheral neuropathy induced by EV is attributed to the non-specific uptake of EV by peripheral nerves and the release of MMAE, which disrupte MTs and leads to neurodegeneration (36). When occurring with peripheral neuropathy events in EV therapy, neurotrophic drugs could be used for symptomatic treatment.

Aside from these, we discovered some positive PTs that are not listed on the drug label and refer to gastrointestinal, hepatic, and pulmonary events. First, the gastrointestinal events notably including ileus, intestinal obstruction, gastrointestinal perforation, mechanical ileus, and ileus paralytic, with intestinal obstruction accounting for a large proportion. Notably, disitamab vedotin (RC48), another ADC drug conjugated with MMAE, has been reported to induce intestinal obstruction (37, 38). The plant neurotoxicity of MMAE was thought to be a cause of intestinal obstruction. Second, for hepatic events, we detected that alanine aminotransferase, aspartate aminotransferase, gamma-glutamyltransferase, and transaminase signals increased, and hepatic function was abnormal. A pharmacovigilance study, by FAERS data mining, found that there was an association between drug-induced liver injury and EV (39). The continuous monitoring of liver function is essential, and further research is needed to explore the underlying mechanisms. Third, Our analysis found some pulmonary event PTs signal (e.g., pneumonitis, pulmonary toxicity, and immune-mediated lung disease). Wanglong et al. discovered that EV exhibited strong safety signals for interstitial lung disease (40). Thus, there was a potential association between EV and a pulmonary adverse event. Altogether, clinicians should be aware of these significant and unlabeled PTs. Further studies need to comprehensively explore the clinical causation of EV and these significant unlabeled PTs.

The time-to-onset analysis discovered that 9.46% of AE events occurred 24 h after exposure to EV, and 66.05% of AE events occurred within 30 days; only 0.73% of AE events occurred after 1 year. Among the top 10 most frequently reported PTs, neuropathy peripheral had the longest mean onset time and the lowest cumulative percentage of AE reports occurring within 30 days. For the long-term safety data, a study reported that after a median follow-up of approximately 2 years, the tolerability of EV was consistent with findings for the interim/primary analysis, no new safety signals were observed (23). Some EV-related AEs may be predictable and possibly preventable through informed patient selection and the increased monitoring of high-risk patients. Thus, we emphasize the monitoring and management of AEs on the day of medication and over the following 30 days. Otherwise, late-onset AEs, like peripheral neuropathy, also need to be taken seriously.

Some limitations of this study must be acknowledged. First, owing to the inherent limitations of FAERS, the presence of bias in this study was unavoidable and cannot be eliminated. Second, the FAERS database lacks information on the severity of AEs, limiting our ability to conduct additional research on the AE grade. Third, the disproportionality analyses could not draw a causal relationship between the drug and AEs, and lacked a clinically conducted causality assessment. More comprehensive clinical studies are required to confirm our findings and explore the underlying mechanism. Despite these limitations, the present study can provide insight into the nature of future studies investigating a possible causal link with EV.

## 5 Conclusions

The pharmacovigilance analysis systematically quantified the safety profile of EV based on real-world AE reports from the FAERS. Our study highlights the importance of the awareness of EV-related skin reactions, especially severe skin reactions such as SJS/TEN. The frequent AEs (e.g., peripheral neuropathy, decreased appetite, malaise, and pyrexia) and unlabeled AEs (e.g., gastrointestinal, hepatic, and pulmonary events) also need to be monitored. Most AEs occurred within 30 days; therefore, thus it is crucial to enhance prevention and proper management for patients undergoing EV therapy. Further studies on the potential mechanisms and preventive measures for EV-related AEs are needed.

## Data Availability

The original contributions presented in the study are included in the article/**Supplementary Material**. Further inquiries can be directed to the corresponding authors.
