# Supplementary material for: Post-marketing drug safety surveillance of enfortumab vedotin: an observational pharmacovigilance study based on a real-world database
Source: Front Immunol. 2024 Aug 20;15:1397692. doi: 10.3389/fimmu.2024.1397692 (PMC11372787; doi:10.3389/fimmu.2024.1397692)
Supplement: Supplementary file 1 [file DataSheet1.docx]

**Post-marketing drug safety surveillance of Enfortumab Vedotin: An observation,pharmacovigilance study based on real-world database**

**Supplementary material**

**Supplementary Figure 1:The Venn diagram of significant disproportionality PTs for the four major algorthms.**

**
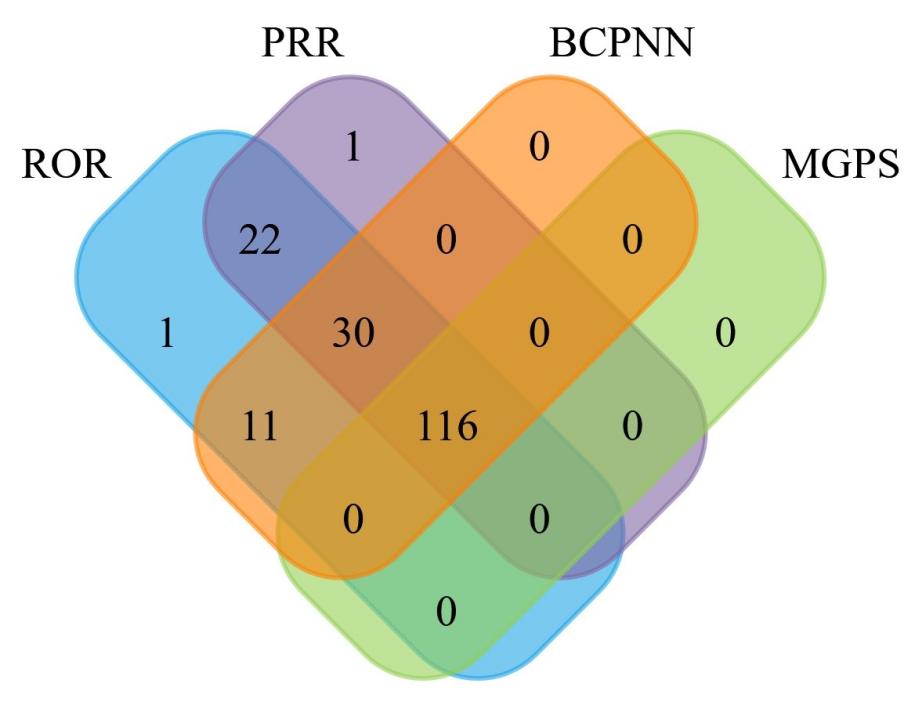
**

**Supplementary Table 1:Two-by-two contingency table**

|  | **Drug exposure** | **No drug exposure** | **Total** |
| --- | --- | --- | --- |
| **Adverse event occurred** | a | c | a+c |
| **No adverse event occurred** | b | d | b+d |
| **Total** | a+b | c+d | a+b+c+d |

a, number of reports containing both the target drug and target adverse event;

b, number of reports containing other adverse event of the target drug;

c, number of reports containing the target aadverse event of other drugs;

d, number of reports containing other drugs and other adverse event.

**Supplementary Table 2:Four major algorthms used for disproportionality analysis**

| **Algorithms** | **Equation** | **Criteria** |
| --- | --- | --- |
| ROR | ROR = (a/b)/(c/d)  95% CI = e^ln(ROR)^ ^± 1.96(1/a + 1/b + 1/c + 1/d)^0.5^ | 95% CI > 1, *N* ≥ 3 |
| PRR | PRR=[a/(a+b)]/[c/(c+d)]  χ^2^=[(ad-bc)^2^](a+b+c+d)/[(a+b)(c+d)(a+c)(b+d)] |  |
| MGPS | EBGM = a(a + b + c + d)/[(a + c)(a + b)]  95%CI=e^ln(EBGM)±1.96(1/a+1/b+1/c+1/d)^0.5^ | EBGM05 > 2 |
| BCPNN | IC = log_2_a(a + b + c + d)/[(a + c)(a + b)]  95%CI= E(IC) ± 2V(IC)^0.5 | IC025 > 0 |

N, the number of reports;

95%CI, 95% confidence interval; χ2, chi-squared; IC, information component; IC025, the lower limit of 95% CI of the IC; E(IC),the IC expectations; V(IC), the variance of IC; EBGM, empirical Bayesian geometric mean; EBGM05, the lower limit of 95% CI of EBGM

**Supplementary Table3: Signal strength for EV based on SMQ level in FAERS.**

| **SMQ** | **N** | **ROR(95%CI)** | **PRR(**χ2) | **EBGM(EBGM05)** | **IC(IC25)** |
| --- | --- | --- | --- | --- | --- |
| Hypersensitivity | 748 | 4.23(3.92-4.56) | 3.90(1653.81) | 3.90(3.61) | 1.96(1.85) |
| Haematopoietic cytopenias | 429 | 4.32(3.92-4.76) | 4.13(1028.52) | 4.12(3.74) | 2.04(1.89) |
| **Peripheral neuropathy** | **270** | **14.25(12.61-16.09)** | **13.76(3186.51)** | **13.69(12.12)** | **3.78(3.53)** |
| Hepatic disorders | 232 | 3.03(2.66-3.45) | 2.97(305.48) | 2.97(2.60) | 1.57(1.36) |
| Oropharyngeal disorders | 208 | 3.14(2.73-3.60) | 3.08(294.06) | 3.08(2.68) | 1.62(1.40) |
| Hyperglycaemia/new onset diabetes mellitus | 207 | 5.18(4.51-5.95) | 5.06(676.79) | 5.05(4.40) | 2.34(2.11) |
| Agranulocytosis | 196 | 7.58(6.57-8.73) | 7.40(1085.94) | 7.38(6.41) | 2.88(2.63) |
| **Severe cutaneous adverse reactions** | **191** | **17.64(15.27-20.38)** | **17.21(2901.31)** | **17.10(14.81)** | **4.10(3.77)** |
| Gastrointestinal perforation, ulceration, haemorrhage or obstruction | 156 | 2.42(2.06-2.84) | 2.39(127.04) | 2.39(2.04) | 1.26(1.01) |
| Taste and smell disorders | 149 | 9.97(8.48-11.73) | 9.79(1173.91) | 9.76(8.29) | 3.29(2.97) |
| Interstitial lung disease | 91 | 6.80(5.53-8.37) | 6.73(443.87) | 6.72(5.46) | 2.75(2.36) |
| Sepsis | 67 | 3.40(2.67-4.33) | 3.38(112.33) | 3.38(2.65) | 1.75(1.35) |
| Biliary disorders | 50 | 4.18(3.17-5.53) | 4.16(120.15) | 4.16(3.15) | 2.06(1.56) |
| Dehydration | 45 | 3.51(2.62-4.71) | 3.50(80.33) | 3.50(2.61) | 1.81(1.30) |
| Conjunctival disorders | 41 | 4.17(3.06-5.67) | 4.15(97.97) | 4.14(3.05) | 2.05(1.50) |
| Lacrimal disorders | 38 | 3.64(2.64-5.01) | 3.62(72.22) | 3.62(2.63) | 1.86(1.30) |
| Hyponatraemia/SIADH | 32 | 3.65(2.58-5.17) | 3.64(61.19) | 3.63(2.57) | 1.86(1.25) |
| **Retroperitoneal fibrosis** | **25** | **13.07(8.81-19.37)** | **13.03(276.23)** | **12.96(8.75)** | **3.70(2.58)** |
| Eosinophilic pneumonia | 22 | 6.45(4.24-9.80) | 6.43(100.66) | 6.42(4.22) | 2.68(1.77) |
| Acute pancreatitis | 21 | 3.32(2.16-5.09) | 3.31(33.84) | 3.31(2.15) | 1.73(0.96) |
| Opportunistic infections | 17 | 4.79(2.98-7.72) | 4.78(50.81) | 4.78(2.97) | 2.26(1.30) |
| Extravasation events (injections, infusions and implants) | 16 | 5.34(3.27-8.73) | 5.33(56.20) | 5.32(3.26) | 2.41(1.38) |

**Supplementary Table4: The distribution of positive signals of EV based on preferred terms categorized by system organ classes.**

| **SOC** | **report number** | **percent(%)** | **PTs number** | **percent(%)** |
| --- | --- | --- | --- | --- |
| Skin and subcutaneous tissue disorders | 1189 | 36.29 | 31 | 26.72 |
| Metabolism and nutrition disorders | 435 | 13.28 | 14 | 12.07 |
| Nervous system disorders | 434 | 13.25 | 7 | 6.03 |
| Blood and lymphatic system disorders | 372 | 11.35 | 10 | 8.62 |
| General disorders and administration site conditions | 297 | 9.07 | 9 | 7.76 |
| Investigations | 122 | 3.72 | 7 | 6.03 |
| Gastrointestinal disorders | 113 | 3.45 | 12 | 10.34 |
| Infections and infestations | 100 | 3.05 | 9 | 7.76 |
| Respiratory, thoracic and mediastinal disorders | 93 | 2.84 | 5 | 4.31 |
| Hepatobiliary disorders | 71 | 2.17 | 5 | 4.31 |
| Eye disorders | 31 | 0.95 | 3 | 2.59 |
| Endocrine disorders | 10 | 0.31 | 1 | 0.86 |
| Vascular disorders | 6 | 0.18 | 2 | 1.72 |
| Renal and urinary disorders | 3 | 0.09 | 1 | 0.86 |

**Supplementary Table5: Signal strength for Enfortumab vedotin based on preferred terms categorized by system organ classes.**

| SOC | PT | **N** | **ROR(95%CI)** | **PRR** | **EBGM(EBGM05)** | **IC(IC25)** |
| --- | --- | --- | --- | --- | --- | --- |
| Skin and subcutaneous tissue disorders | rash | 327 | 6.61(5.92,7.39) | 6.36(1484.76) | 6.35(5.68) | 2.67(2.48) |
|  | alopecia | 133 | 5.90(4.97,7.01) | 5.81(530.36) | 5.80(4.89) | 2.54(2.23) |
|  | pruritus | 133 | 3.10(2.62,3.69) | 3.07(186.11) | 3.06(2.58) | 1.62(1.34) |
|  | skin disorder | 111 | 26.05(21.58,31.45) | 25.67(2607.03) | 25.42(21.06) | 4.67(4.11) |
|  | stevens-johnson syndrome* | 60 | 38.26(29.62,49.43) | 37.96(2127.65) | 37.41(28.96) | 5.23(4.18) |
|  | toxic epidermal necrolysis* | 56 | 37.42(28.71,48.76) | 37.14(1941.10) | 36.61(28.10) | 5.19(4.11) |
|  | drug eruption | 37 | 18.79(13.59,25.99) | 18.70(615.56) | 18.57(13.43) | 4.22(3.20) |
|  | skin toxicity | 34 | 48.86(34.77,68.65) | 48.64(1556.41) | 47.73(33.97) | 5.58(3.86) |
|  | skin exfoliation | 34 | 3.21(2.29,4.49) | 3.20(51.30) | 3.19(2.28) | 1.67(1.10) |
|  | blister | 30 | 4.81(3.36,6.89) | 4.79(89.97) | 4.79(3.34) | 2.26(1.57) |
|  | rash pruritic | 29 | 5.11(3.55,7.36) | 5.09(95.25) | 5.08(3.53) | 2.35(1.63) |
|  | dermatitis bullous | 27 | 39.00(26.65,57.08) | 38.87(981.03) | 38.29(26.16) | 5.26(3.49) |
|  | rash erythematous | 25 | 5.39(3.64,7.98) | 5.37(88.87) | 5.36(3.62) | 2.42(1.63) |
|  | rash maculo-papular | 22 | 8.97(5.90,13.64) | 8.94(154.74) | 8.92(5.86) | 3.16(2.13) |
|  | skin discolouration* | 19 | 3.73(2.38,5.86) | 3.73(37.85) | 3.72(2.37) | 1.90(1.06) |
|  | skin reaction | 18 | 13.69(8.61,21.77) | 13.66(210.07) | 13.59(8.55) | 3.76(2.37) |
|  | pigmentation disorder* | 11 | 14.44(7.98,26.12) | 14.42(136.57) | 14.34(7.92) | 3.84(1.93) |
|  | rash papular | 9 | 4.27(2.22,8.21) | 4.26(22.45) | 4.26(2.21) | 2.09(0.77) |
|  | exfoliative rash | 8 | 34.04(16.94,68.41) | 34.00(252.85) | 33.56(16.70) | 5.07(1.89) |
|  | dermatitis exfoliative generalised | 8 | 14.08(7.03,28.22) | 14.07(96.57) | 13.99(6.98) | 3.81(1.55) |
|  | skin erosion* | 7 | 17.52(8.33,36.87) | 17.51(108.21) | 17.39(8.27) | 4.12(1.49) |
|  | acute generalised exanthematous pustulosis* | 7 | 7.38(3.51,15.50) | 7.37(38.45) | 7.35(3.50) | 2.88(1.01) |
|  | toxic erythema of chemotherapy | 6 | 146.69(64.39,334.22) | 146.58(819.75) | 138.56(60.82) | 7.11(1.62) |
|  | symmetrical drug-related intertriginous and flexural exanthema* | 6 | 39.66(17.70,88.86) | 39.63(222.41) | 39.03(17.42) | 5.29(1.50) |
|  | lichenoid keratosis* | 6 | 26.98(12.07,60.33) | 26.96(148.39) | 26.68(11.93) | 4.74(1.42) |
|  | rash vesicular | 6 | 12.24(5.49,27.32) | 12.23(61.60) | 12.18(5.46) | 3.61(1.13) |
|  | epidermal necrosis | 5 | 52.24(21.54,126.68) | 52.20(246.01) | 51.16(21.10) | 5.68(1.26) |
|  | cutaneous symptom | 5 | 50.97(21.02,123.58) | 50.94(239.92) | 49.95(20.60) | 5.64(1.25) |
|  | dermatitis exfoliative | 4 | 25.49(9.52,68.28) | 25.48(93.14) | 25.24(9.42) | 4.66(0.81) |
|  | leukoderma* | 3 | 109.45(34.44,347.79) | 109.40(308.81) | 104.88(33.01) | 6.71(0.48) |
|  | rash morbilliform | 3 | 10.19(3.28,31.68) | 10.19(24.76) | 10.15(3.27) | 3.34(0.18) |
| Nervous system disorders | neuropathy peripheral | 226 | 19.32(16.91,22.06) | 18.76(3777.58) | 18.63(16.31) | 4.22(3.92) |
|  | taste disorder | 121 | 27.54(22.99,32.99) | 27.11(3011.80) | 26.83(22.40) | 4.75(4.20) |
|  | hypoaesthesia | 49 | 3.25(2.45,4.30) | 3.23(75.59) | 3.23(2.44) | 1.69(1.22) |
|  | peripheral sensory neuropathy | 16 | 25.86(15.80,42.34) | 25.81(377.70) | 25.56(15.61) | 4.68(2.68) |
|  | polyneuropathy | 10 | 6.26(3.36,11.64) | 6.25(44.00) | 6.24(3.35) | 2.64(1.21) |
|  | peripheral motor neuropathy | 9 | 56.54(29.19,109.51) | 56.47(479.67) | 55.26(28.53) | 5.79(2.18) |
|  | chronic inflammatory demyelinating polyradiculoneuropathy* | 3 | 15.97(5.13,49.69) | 15.96(41.80) | 15.86(5.10) | 3.99(0.30) |
| Blood and lymphatic system disorders | myelosuppression | 99 | 17.53(14.37,35.64) | 17.31(1512.06) | 17.20(14.09) | 4.10(3.60) |
|  | neutropenia | 82 | 4.33(3.49,5.39) | 4.30(207.57) | 4.29(3.45) | 2.10(1.73) |
|  | anaemia | 69 | 3.54(2.79,4.48) | 3.51(124.25) | 3.51(2.77) | 1.81(1.41) |
|  | febrile neutropenia | 55 | 6.61(5.07,8.62) | 6.57(259.09) | 6.55(5.02) | 2.71(2.19) |
|  | leukopenia | 20 | 3.74(2.41,5.80) | 3.73(39.98) | 3.73(2.40) | 1.90(1.09) |
|  | pancytopenia | 19 | 3.32(2.12,5.21) | 3.31(30.67) | 3.31(2.11) | 1.73(0.92) |
|  | cytopenia | 10 | 4.77(2.56,8.87) | 4.76(29.67) | 4.75(2.56) | 2.25(0.95) |
|  | disseminated intravascular coagulation* | 8 | 7.29(3.64,14.59) | 7.28(43.22) | 7.26(3.63) | 2.86(1.13) |
|  | bone marrow failure | 7 | 5.06(2.41,10.62) | 5.06(22.73) | 5.05(2.40) | 2.34(0.72) |
|  | febrile bone marrow aplasia | 3 | 7.07(2.28,21.96) | 7.07(15.59) | 7.05(2.27) | 2.82(0.04) |
| Metabolism and nutrition disorders | decreased appetite | 162 | 6.13(5.25,7.17) | 6.02(678.94) | 6.01(5.14) | 2.59(2.31) |
|  | hyperglycaemia | 102 | 29.28(24.06,35.64) | 28.89(2716.74) | 28.58(23.48) | 4.84(4.21) |
|  | dehydration | 43 | 3.49(2.59,4.71) | 3.48(75.95) | 3.47(2.57) | 1.80(1.28) |
|  | diabetes mellitus | 27 | 3.52(2.41,5.14) | 3.51(48.50) | 3.51(2.40) | 1.81(1.14) |
|  | hyponatraemia | 24 | 4.18(2.80,6.24) | 4.17(57.71) | 4.16(2.79) | 2.06(1.31) |
|  | diabetic ketoacidosis | 20 | 8.16(5.26,12.67) | 8.14(124.98) | 8.12(5.23) | 3.02(1.97) |
|  | hypercalcaemia* | 13 | 9.27(5.37,15.98) | 9.25(95.33) | 9.22(5.35) | 3.20(1.76) |
|  | hypocalcaemia* | 11 | 5.62(3.11,10.17) | 5.62(41.67) | 5.61(3.10) | 2.49(1.18) |
|  | appetite disorder | 10 | 12.02(6.45,22.38) | 12.00(100.41) | 11.95(6.42) | 3.58(1.71) |
|  | hypophosphataemia | 7 | 7.64(3.64,16.06) | 7.64(40.27) | 7.62(3.63) | 2.93(1.04) |
|  | glucose tolerance impaired | 5 | 8.04(3.34,19.35) | 8.03(30.70) | 8.01(3.33) | 3.00(0.70) |
|  | hyperuricaemia | 4 | 9.32(3.49,24.89) | 9.32(29.60) | 9.29(3.48) | 3.22(0.51) |
|  | cachexia* | 4 | 7.30(2.74,19.49) | 7.30(21.68) | 7.28(2.73) | 2.86(0.40) |
|  | insulin resistance* | 3 | 11.78(3.79,36.63) | 11.78(29.45) | 11.73(3.77) | 3.55(0.22) |
| General disorders and administration site conditions | malaise | 121 | 2.71(2.27,3.25) | 2.69(128.76) | 2.68(2.24) | 1.42(1.14) |
|  | pyrexia | 117 | 3.07(2.56,3.68) | 3.04(160.47) | 3.03(2.53) | 1.60(1.31) |
|  | multiple organ dysfunction syndrome* | 22 | 5.03(3.31,7.65) | 5.02(70.76) | 5.01(3.30) | 2.33(1.49) |
|  | mucosal inflammation* | 11 | 3.95(2.19,7.15) | 3.95(24.21) | 3.95(2.18) | 1.98(0.83) |
|  | infusion site extravasation | 8 | 7.39(3.69,14.79) | 7.38(43.99) | 7.36(3.68) | 2.88(1.14) |
|  | performance status decreased | 7 | 18.40(8.75,38.72) | 18.39(114.25) | 18.26(8.68) | 4.19(1.51) |
|  | extravasation | 4 | 12.40(4.64,33.13) | 12.40(41.70) | 12.34(4.62) | 3.63(0.62) |
|  | physical deconditioning | 4 | 8.90(3.33,23.77) | 8.90(27.95) | 8.87(3.32) | 3.15(0.49) |
|  | mucosal disorder* | 3 | 12.73(4.09,39.61) | 12.73(32.26) | 12.67(4.07) | 3.66(0.24) |
| Investigations | neutrophil count decreased | 54 | 9.88(7.56,12.92) | 9.82(426.31) | 9.78(7.48) | 3.29(2.68) |
|  | aspartate aminotransferase increased* | 20 | 4.24(2.74,6.59) | 4.24(49.38) | 4.23(2.73) | 2.08(1.24) |
|  | alanine aminotransferase increased* | 20 | 3.48(2.24,5.40) | 3.47(35.19) | 3.47(2.24) | 1.79(1.00) |
|  | transaminases increased* | 10 | 3.96(2.13,7.37) | 3.96(22.08) | 3.95(2.12) | 1.98(0.77) |
|  | gamma-glutamyltransferase increased* | 8 | 4.57(2.28,9.15) | 4.57(22.27) | 4.56(2.28) | 2.19(0.74) |
|  | kl-6 increased* | 6 | 143.90(63.19,372.72) | 143.78(804.77) | 136.07(59.75) | 7.09(1.62) |
|  | blood uric acid increased | 4 | 6.33(2.37,16.89) | 6.33(17.89) | 6.31(2.37) | 2.66(0.32) |
| Hepatobiliary disorders | hepatic function abnormal* | 46 | 11.01(8.24,14.73) | 10.95(414.36) | 10.91(8.16) | 3.45(2.75) |
|  | cholangitis* | 9 | 12.76(6.62,24.57) | 12.74(96.92) | 12.68(6.59) | 3.67(1.63) |
|  | cholecystitis* | 8 | 8.26(4.13,16.55) | 8.26(50.86) | 8.23(4.11) | 3.04(1.23) |
|  | immune-mediated hepatitis* | 5 | 13.80(5.73,33.25) | 13.80(59.02) | 10.91(8.16) | 3.45(2.75) |
|  | bile duct stone* | 3 | 9.76(3.14,30.32) | 9.75(23.48) | 9.72(3.13) | 3.28(0.16) |
| Respiratory, thoracic and mediastinal disorders | interstitial lung disease | 41 | 7.36(5.41,10.01) | 7.33(223.48) | 7.31(5.37) | 2.87(2.22) |
|  | pneumonitis* | 22 | 6.45(4.24,9.80) | 6.43(100.66) | 6.42(4.22) | 2.68(1.77) |
|  | pulmonary toxicity* | 18 | 18.61(11.70,29.60) | 18.56(296.94) | 18.43(11.59) | 4.20(2.60) |
|  | aspiration* | 7 | 6.78(3.23,14.25) | 6.78(34.38) | 6.76(3.22) | 2.76(0.95) |
|  | immune-mediated lung disease* | 5 | 17.76(7.37,42.81) | 17.74(78.46) | 17.63(7.31) | 4.14(1.04) |
| Infections and infestations | sepsis | 36 | 3.05(2.20,4.23) | 3.04(49.19) | 3.03(2.19) | 1.60(1.05) |
|  | pyelonephritis* | 17 | 17.53(10.88,28.27) | 17.50(262.60) | 17.38(10.78) | 4.12(2.50) |
|  | septic shock* | 17 | 3.54(2.20,5.70) | 3.53(30.83) | 3.53(2.19) | 1.82(0.95) |
|  | pneumonia aspiration | 13 | 4.95(2.87,8.53) | 4.94(40.82) | 4.94(2.86) | 2.30(1.17) |
|  | oesophageal candidiasis* | 4 | 10.51(3.94,28.07) | 10.51(34.26) | 10.47(3.92) | 3.39(0.56) |
|  | staphylococcal sepsis* | 4 | 9.87(3.70,26.36) | 9.87(31.75) | 9.83(3.68) | 3.30(0.53) |
|  | muscle abscess* | 3 | 46.33(14.78,145.18) | 46.31(130.60) | 45.49(14.52) | 5.51(0.45) |
|  | psoas abscess* | 3 | 45.49(14.52,142.53) | 45.47(128.17) | 44.68(14.26) | 5.48(0.45) |
|  | escherichia urinary tract infection | 3 | 6.77(2.18,21.02) | 6.76(14.70) | 6.75(2.17) | 2.75(0.02) |
| Gastrointestinal disorders | stomatitis* | 25 | 3.38(2.28,5.01) | 3.37(41.68) | 3.37(2.27) | 1.75(1.06) |
|  | ileus* | 18 | 17.34(10.90,27.58) | 17.30(274.60) | 17.19(10.812) | 4.10(2.55) |
|  | intestinal obstruction* | 16 | 3.87(2.37,6.31) | 3.86(33.86) | 3.85(2.36) | 1.95(1.02) |
|  | immune-mediated enterocolitis | 11 | 17.26(9.53,31.24) | 17.23(167.09) | 17.12(9.46) | 4.10(2.03) |
|  | pancreatitis acute | 9 | 4.26(2.21,8.19) | 4.26(22.38) | 4.25(2.21) | 2.09(0.77) |
|  | enterocolitis | 7 | 9.53(4.54,20.03) | 9.53(53.22) | 9.49(4.52) | 3.25(1.18) |
|  | duodenal ulcer | 6 | 10.33(4.63,23.05) | 10.33(50.34) | 10.29(4.61) | 3.36(1.05) |
|  | mechanical ileus* | 5 | 59.95(24.69,145.58) | 59.91(282.90) | 58.54(24.11) | 5.87(1.27) |
|  | gastrointestinal perforation* | 5 | 11.92(4.95,28.71) | 11.91(49.76) | 11.86(4.93) | 3.57(0.90) |
|  | small intestinal perforation | 4 | 22.04(8.23,58.98) | 22.02(79.58) | 21.84(8.16) | 4.45(0.78) |
|  | ileus paralytic* | 4 | 11.03(4.13,29.46) | 11.02(36.30) | 10.98(4.11) | 3.46(0.58) |
|  | gastric ulcer haemorrhage* | 3 | 6.50(2.09,20.20) | 6.50(13.93) | 6.49(2.09) | 2.70(0.01) |
| Eye disorders | dry eye | 25 | 4.00(2.70,5.93) | 3.99(55.99) | 3.99(2.69) | 1.99(1.27) |
|  | ocular toxicity | 3 | 22.68(7.28,70.69) | 22.67(61.58) | 22.48(7.21) | 4.49(0.37) |
|  | keratitis | 3 | 9.05(2.91,28.14) | 9.05(21.41) | 9.02(2.90) | 3.17(0.14) |
| Endocrine disorders | adrenal insufficiency* | 10 | 5.76(3.10,10.72) | 5.76(39.22) | 5.75(3.09) | 2.52(1.13) |
| Vascular disorders | thrombophlebitis migrans* | 3 | 40.38(12.90,126.38) | 40.37(113.36) | 39.75(12.70) | 5.31(0.44) |
|  | venous thrombosis limb* | 3 | 14.01(4.50,43.59) | 14.01(36.03) | 13.93(4.48) | 3.80(0.27) |
| Renal and urinary disorders | nephritis* | 3 | 7.91(2.55,24.57) | 7.90(18.04) | 7.88(2.54) | 2.98(0.09) |

SOCs = system organ classes, PT = preferred terms, PRR = proportional reporting ratio, χ2 = chi-square. a Adverse events which were not mentioned in the instruction of enfortumab vedotin.

*PT not list in the label of EV
